# Long-term outcome of ^177^Lu-PSMA-617 radioligand therapy in heavily pre-treated metastatic castration-resistant prostate cancer patients

**DOI:** 10.1371/journal.pone.0251375

**Published:** 2021-05-10

**Authors:** Madhav Prasad Yadav, Sanjana Ballal, Ranjit Kumar Sahoo, Madhavi Tripathi, Nishikant Avinash Damle, Shamim Ahmed Shamim, Rakesh Kumar, Amlesh Seth, Chandrasekhar Bal

**Affiliations:** 1 Department of Nuclear Medicine, Thyroid Clinic, AIIMS, Ansari Nagar, New Delhi, India; 2 Department of Medical Oncology, IRCH, AIIMS, Ansari Nagar, New Delhi, India; 3 Department of Urology, AIIMS, Ansari Nagar, New Delhi, India; University of Toronto, CANADA

## Abstract

**Objective:**

Investigators have extensively explored the short-term safety and efficacy data on ^177^Lu-PSMA-617 radioligand therapy (RLT) in mCRPC patients. However, scarce literature is reported on the long-term outcome of these patients. The current goal of this study is focused on the long-term outcome of mCRPC patients treated with ^177^Lu-PSMA-617 RLT.

**Methods:**

Among 135 patients, 121 mCRPC patients fulfilled the eligibility criteria and were included in the final analysis. Patients received a median of 3 cycles of ^177^Lu-PSMA-617 RLT at 6 to 12-week intervals. Primary endpoint included overall survival (OS) and secondary endpoints involved progression-free survival (PFS), predictive factors of OS and PFS, PSA response rate, molecular response, clinical response, and toxicity assessment.

**Results:**

The median administered cumulative activity was 20 GBq (3.7–37 GBq). The median follow-up duration was 36 months (6–72 months). The estimated median PFS and OS were 12 months (mo) (95% CI: 10.3–13 mo) and 16 mo (95% CI: 13–17 mo), respectively. Any PSA decline and PSA decline >50% was achieved in 73% and 61% of the patients, respectively. Multivariate analysis revealed only failure to achieve >50% PSA decline as a significant factor associated with a poor PFS. Prognostic factors associated with reduced OS included, failure to experience >50% PSA decline, heavily pre-treated patient cohort who received >2 lines of prior treatment options, and patient sub-group treated with ≥2 lines of chemotherapy. Patients re-treated with additional treatment options after attaining ^177^Lu-PSMA refractory disease showed a remarkably prolonged OS. A significant clinical benefit was achieved post ^177^Lu-PSMA-617 RLT. The most common toxicities observed were fatigue (34.7%), followed by nausea (33%), and dry mouth (24.7%).

**Conclusion:**

The current study supports the short-term safety and efficacy results of high response rates, prolonged PFS and OS, improved quality of life, and low treatment-related toxicities in patients treated with ^177^Lu-PSMA-617 radioligand therapy.

## Introduction

Radioligand therapy (RLT) with ^177^Lu-PSMA-617 is a promising option in treating metastatic castration-resistant prostate cancer (mCRPC). A meta-analysis consolidated the results of several retrospective studies [1], including a prospective phase II Australian clinical trial, and has confirmed the efficacy of ^177^Lu-PSMA-617 therapy in mCRPC patients [2].

Following promising clinical and biochemical responses from the phase II results on ^177^Lu-PSMA-617 RLT, a randomized phase III study, the VISION trial (ClinicalTrials.gov, NCT03511664) is underway in mCRPC patients. If the VISION trial proves successful, RLT will enter the therapeutic algorithm to manage advanced prostate cancer.

Radioligand therapy using ^177^Lu-PSMA-617 has improved the overall survival (OS) and progression-free survival (PFS) in heavily pre-treated mCRPC patients. A phase II clinical trial on ^177^Lu-PSMA-617 molecular radiotherapy in patients with progressive mCRPC was conducted at the University of California Los Angeles, USA, and Excel Diagnostics & Nuclear Oncology Centre (Houston, TX, USA) (NCT03042312). According to their post hoc analysis, the median OS after ^177^Lu-PSMA-617 RLT was 14.8 months (mo) [3].

All the studies conducted to date have focused on short-term preliminary results including safety and efficacy data with limited long-term follow-up data [4]. In our previous short-term study in 90 extensively pre-treated mCRPC patients, the PSA response rate was 45.5% [5]. However, no long-term outcome data have come forth to validate the short-term data or understand the pattern of response after the ^177^Lu-PSMA-617 treatment. Moreover, it is also essential to know the treatment paradigm after progressing on ^177^Lu-PSMA-617 therapy. In this context, a pilot phase II study from the Australian New Zealand trial group has reported long-term outcomes of retreatment with ^177^Lu-PSMA-617 in an expanded cohort of 50 mCRPC patients which includes 30 patients who were previously enrolled in the phase II trial. The evidence from their study suggests higher response rates in patients re-challenged with ^177^Lu-PSMA-617 compared to other systemic therapies [4].

Despite the encouraging results, the long-term data is crucial to decide if ^177^Lu-PSMA-617 RLT therapy has a sustained response rate. This study objective was to detail the single-institution-based, long-term follow-up and outcome of an extended cohort of patients who received ^177^Lu-PSMA-617 therapy. This study identifies the prognostic factors associated with PFS and OS, gives an overview of the real-world clinical scenario of these patients after ^177^Lu-PSMA-617 therapy, the patterns of response, alternative subsequent anti-cancer systemic treatments following ^177^Lu-PSMA-617 therapy, and validate the findings with the short-term reports.

## Materials and methods

### Patients

The study protocol was approved by the All India Institute of Medical Sciences, institute ethics committee [Ref. No IEC-517] and conducted by the ethical standards declared by the Helsinki Convention. This study was funded by the Indian Council of Medical Research and registered on the Clinical Trial Registry-India (CTRI Ref No: CTRI/06/006998).

Patients were extensively discussed within the multidisciplinary tumor board following which they were referred for RLT to the Department of Nuclear Medicine. Patients were screened for the following eligibility criteria before the recruitment and written informed consent was signed by the patients.

### Eligibility criteria for enrollment in the study

Essential criteria included pathologically confirmed prostatic adenocarcinoma, documented mCRPC status, progressive disease on standard treatment options including taxane-based chemotherapy, first-line and or second-line anti-androgen treatment, and/or androgen inhibitor therapies, patients on concomitant systemic treatments, documented disease progression on ^68^Ga-PSMA PET/CT scan obtained within 28 days before the beginning of ^177^Lu-PSMA-617 RLT with PSMA expression in lesions greater than the liver for soft tissue lesions and uptake greater than vertebra for the skeletal metastases and ECOG performance status up to 4.

Patients were excluded if they had haemoglobin < 9 g/dL, absolute neutrophil count (ANC) <1.5 x 109 /L, Platelets <60 x 109 /L, bilirubin >1.5 X upper normal limit (UNL), GFR < 40mL/min/1.73m^2^ BSA), patients with serious comorbidities and medical conditions such as congestive heart failure, sensitivity to drugs, cord compression and those who denied giving written informed consent were excluded from the study.

### Final patient recruitment

Among 135 consecutive mCRPC patients screened for eligibility, 14 patients dropped out for the following reasons: four had neuroendocrine differentiation of prostate cancer, six demonstrated low PSMA avidity on baseline ^68^Ga-PSMA-11 PET/CT scan, three patients had grade III haematological toxicity associated with previous therapies, and one patient did not follow-up and dropped out after the first cycle of ^177^Lu-PSMA-617 therapy on self-consent. Finally, 121 patients with a median age of 67 years who fulfilled the mandate criteria were included in the analysis.

### Diagnostic ^68^Ga-PSMA-11 PET/CT

As a prerequisite, all patients underwent a pre-therapy diagnostic ^68^Ga-PSMA-11 PET/CT scans using ^68^Ga-PSMA-HBED-CC to ensure the presence of PSMA overexpression in a majority of the lesions.

### Laboratory assessments

Before every cycle of ^177^Lu-PSMA-617 RLT, complete blood counts (CBC), kidney function tests (KFT), glomerular filtration rate (GFR), liver function tests (LFT), and serum prostate specific antigen (sPSA) levels were documented in all patients.

### ^177^Lu-PSMA-617 infusion

During the preliminary phase of the study when there was no pharmacokinetic data available, the initial 26 patients were treated adopting a single-day kidney protection protocol. However, the early findings of our dosimetry report [6] showed no role of amino acid infusion for kidney protection in ^177^Lu-PSMA-617 RLT. Hence, in the remaining 95 patients, only ^177^Lu-PSMA-617 was diluted in 30 mL normal saline (0.9%), and administered by slow intravenous infusion over 5–10 minutes.

### Dosing of ^177^Lu-PSMA-617

A range of 1.11 to 7.8 GBq (30–210 mCi) of ^177^Lu-PSMA-617 was administered per cycle in the patients. Due to the lack of evidence on the toxicity from ^177^Lu-PSMA-617 RLT, at the initial phase of recruitment in 26 patients, we adopted a dose-escalation protocol which included dosages starting from 1.11 GBq and was gradually escalated up to 5.5 GBq of ^177^Lu-PSMA-617 RLT. Among the 26 patients, 4, 13, 7, and 2 patients received 1.11, 1.85, 3.70 and 5.55 GBq of ^177^Lu-PSMA-617 RLT, respectively. Dosimetry reports from various studies revealed doses up to 9.25 GBq safe for administration without adverse toxicities.

Hence, in the subsequent phase of the study, dosing of ^177^Lu-PSMA-617 RLT ranged between 3.70 to 7.8 GBq at each cycle. The amount of administered activity was based on the extent of metastasis on the ^68^Ga-PSMA-11 PET/CT scan, laboratory parameters, and the ECOG performance status. After the administration of ^177^Lu-PSMA-617, all patients were admitted to the isolation therapy ward for clinical observation and eventually discharged when stable [5].

### Follow-up

Patients were followed-up every 2, 4, and 6–8 weeks (wks) after each cycle of ^177^Lu-PSMA-617 therapy with blood tests to assess for any treatment-related toxicities. After completion of the therapy regimen or discontinuation of treatment, patients were followed regularly at monthly intervals with PSA values and ^68^Ga-PSMA PET/CT at both Nuclear Medicine and Medical Oncology clinics.

## Definitions of outcome endpoints

### Treatment outcome measures

The primary outcome measure included overall survival. Secondary outcome measures involved progression-free survival, factors predicting the OS and PFS, evaluation of the PSA response rate, molecular response assessment, clinical response assessment, and adverse event profile.

#### Survival analysis

*Overall survival*. Time from the commencement of ^177^Lu-PSMA-617 therapy to the death due to any cause or the date of the last contact.

*Progression-free survival*. Defined as the time from the initiation of treatment to the date of documented disease progression (PSA rise >25% from the baseline value, which was further confirmed with a repeat 3-week value). Patients without any tumor progression at the time of analysis were censored at their last tumor evaluation date.

#### Biochemical response

Biochemical response to treatment was assessed as per the Prostate Cancer Working Group 3 criteria (PCWG3) [7].

#### PSA response rate

Defined as the percentage of patients with a PSA reduction of >50% from baseline. Assessed at 2, 4, and 8 wks after every cycle of ^177^Lu-PSMA-617 RLT with repetition at monthly intervals after completion of the ^177^Lu-PSMA-617 treatment regimen.

#### Molecular tumor response

The molecular response was assessed using PERCIST 1 criteria [8].

^68^Ga-PSMA-11 PET/CT scan for the morphological and molecular response was repeated after three cycles of ^177^Lu-PSMA-617 treatment or only if deemed necessary in situations such as subsequent post-therapy scan showed either exceptional response or appearance of the new lesion, new sites of pain development, decrease or doubling of PSA and during the follow-ups after discontinuation or completing the ^177^Lu-PSMA RLT regimen.

#### Clinical response

Clinical response criteria included the assessment of visual analog score (VAS) [9], analgesic score (AS) [9], Karnofsky performance status (KPS) [10], and Eastern Cooperative Oncology Group (ECOG) performance status.

#### Toxicity

Treatment-related adverse events (AEs) were documented as per the National Cancer Institute for Common Toxicity Criteria version 5.0 [11].

### Discontinuation of ^177^Lu-PSMA-617 therapy

Treatment was ceased in the following circumstances: Progression of disease, an early response to treatment which was attained before completion of the treatment regimen, no improvement in the quality of life with no clinical benefit after board discussion with the treating medical oncologist, achieved the maximum administered activity dose limit of ~37 GBq (1000 mCi)

### Statistical analysis

Stata v11.2 statistical software (StataCorp, College Station, TX, USA) and Medcalc version12.5.0 were used to perform the analysis. The D’Agostino-Pearson test was conducted to assess the normality of the data. The normal distributed continuous data were presented as mean, standard deviation, and range. Skewed data were depicted as the median and interquartile range (IQR). Paired-samples t-test (parametric) or Wilcoxon signed-rank test (non-parametric) tests were used to compare the pre and post-therapy parameters.

Kaplan-Meier survival curves were generated, and the Log-rank test was used to compare the OS and PFS of categorized variables. Their median values designated the cut-offs for continuous variables. All variables with P value ≤0.1 on univariate analysis Cox proportional-hazards regression model were included in the multivariate model. For multivariate analysis, the Cox proportional-hazards regression model by stepwise elimination method was carried out to determine the prognostic factors associated with OS and PFS. P-values <0.05 were considered significant.

## Results

Out of 135 mCRPC patients, 121 patients fulfilled the mandate criteria and were included in the analysis. Among the entire series, the first ^177^Lu-PSMA-617 therapy cycle was administered in September 2014, and the last patient was recruited in February 2020. The cut-off date for follow-up was September 2020. According to the reverse Kaplan-Meier censoring method, the median follow-up duration was 36 mo (6–72 mo) from the start of ^177^Lu-PSMA-617 RLT. The baseline characteristics of patients are displayed in Table 1. Fourteen patients had a family history of various cancers and three among them had a family history of prostate cancer. Forty-eight percent belonged to Gleason score 9–10 with 35% having extensive marrow/skeletal involvement and a median ECOG status of 3. Ninety-one percent of (110/121) patients were treated with more than two lines of standard therapies. The remaining 9% had at least two treatment lines before RLT; 83.4% of patients had received docetaxel, 53.7% (65/121) had androgen synthesis inhibitor therapy, and 19% (23/121) received enzalutamide. The median duration of first-line and second-line treatment has been detailed in Table 1.

**Table 1:** Demographic characteristics of patients.

Parameters	Values (N = 121)
Age in years median (IQR)	67 (60.7–72)
Gleason score	
6	3 (2.4%)
7	20 (16.5%)
8	40 (33%)
9–10	58 (48%)
**Primary Treatment**	
Radical Prostatectomy	11 (9%)
Prostatectomy and lymph node dissection	3 (2.4%)
External beam radiotherapy to the pelvis	7 (6%)
**Androgen deprivation therapy (ADT)**	
Surgical castration	63 (52%)
Medical castration (LHRH agonist/antagonist)	19 (16%)
Medical castration followed by surgical castration	39 (32%)
**Anti-androgen therapy**	
First-generation	121 (100%)
**Treatment after attaining castration resistance**	
Second-generation anti-androgen (Enzalutamide)	23 (19%)
**Ketoconazole**	2 (1.6%)
**Androgen synthesis inhibitor (Abiraterone acetate)**	65 (53.7%)
**Chemotherapy**	101 (83.4%)
Docetaxel	90 (89%)
Docetaxel followed by Cabazitaxel	7 (7%)
Docetaxel followed by Cabazitaxel and subsequently with Cyclophosphamide	1 (1%)
Cyclophosphamide	2 (1.6%)
Etoposide + carboplatin	1 (1%)
Palliative external beam radiation therapy	63 (52%)
Bisphosphonates (Zoledronic acid)	96 (79%)
RANK inhibitors (Denosumab)	6 (5%)
Median duration of androgen-deprivation therapy in months (IQR)	12 (4–37)
The median duration of first-generation anti-androgen therapy in months (IQR)	17 (10–29)
The median duration of second-generation anti-androgen therapy in months (IQR)	7 (4–11.5)
The median duration of androgen synthesis inhibitor therapy in months (IQR)	10 (5–14)
The median duration of chemotherapy in months (IQR)	6 (4–10)
**Site and extent of disease on** ^**68**^**Ga-PSMA-11 PET/CT**	
Primary	89 (73%)
Lymph nodes	82 (68%)
Iliac and abdominal	51 (62%)
Thoracic	5 (6%)
Thoracic to iliac	22 (27%)
Neck	4 (5%)
No lymph nodes	39 (32%)
Bone metastases	
≤ 6	11 (9%)
6–20	17 (14%)
>20	50 (41%)
Diffuse/super scan/ Extensive	43 (35.5%)
Lung metastases	9 (7.5%)
Other sites	
Brain	4 (3.3%)
Liver	9 (7.5%)
Adrenals	1 (0.8%)
Baseline median PSA (ng/mL) (median, 25–75% IQR)	222.2 (47–443.2)
Concomitant treatment	31 (25.6%)
Median follow-up after ^177^Lu-PSMA-617 therapy initiation in months (range)	16 (4–46)
Median number of ^177^Lu-PSMA-617 therapy cycles (range)	3 (1–7)
Median cumulative Activity of ^177^Lu-PSMA-617 (MBq) ± SD, (range)	20 GBq (3.7–37 GBq)
	540 mCi (100–1010 mCi)

ADT: Androgen deprivation therapy; AA: Abiraterone acetate, IQR: Interquartile range; PSA: Prostate-specific antigen; SD: Standard deviation, GBq: Gigabecquerel, mCi: millicurie.

PSMA expression was noted in the primary site in 73% of patients. Consistent with the advanced stage of CRPC, all patients in this series presented with skeletal metastases; however, visceral metastases were variable; namely, 68% of patients had lymph node metastases, 7.5% of patients with lung and liver metastases each, and only 3.3% with brain metastases. Thirty-one patients underwent concomitant treatment with a majority (87%; 27/31) who received abiraterone acetate 1gm daily with 5 mg oral prednisolone (AAP). Among the remaining four patients, 3 received enzalutamide (160 mg daily), and one was on a combination of AAP and three cycles of cabazitaxel.

### Dose scheme and treatment cycles

In 2014, we started the ^177^Lu-PSMA-617 RLT when there were no dosimetry reports available for the dose-limiting organs. Thus, the initial 26 patients had a dose-escalation study for dosimetry purposes. In the remaining 95 patients, the ^177^Lu-PSMA-617 administration was an individualized approach. It varied among the patients between 3.70 and 7.78 GBq per cycle.

**Fig 1:**
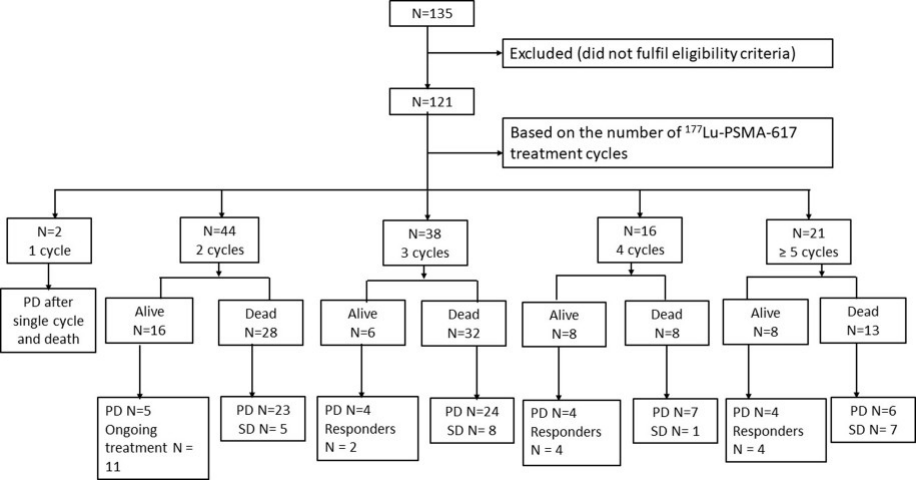
Flow chart depicting the course of treatment and the survival status of the patients categorized according to the number of ^177^Lu-PSMA-617 RLT cycles administered. PD: Progressive disease.

A median of 3 cycles of ^177^Lu-PSMA-617 was administered with the mean cumulative activity of 20 GBq (range, 3.7–37 GBq). In a median time interval of 8 wks (range, 6–12 wks) between each ^177^Lu-PSMA-617 RLT treatment cycle, a total of 386 cycles were administered in 121 mCRPC patients. The number of cycles varied from 2 cycles in 44 (36%), 3 cycles in 38 (32%), and 4 cycles in 16 (13%). Furthermore, 21 (17%) patients received more than four cycles. Only two patients received a single cycle of ^177^Lu-PSMA-617 RLT due to the progression of diseases but were on regular follow-up. The survival status of each patient according to the number of cycles administered is depicted in the flow-chart (Fig 1).

### Biochemical PFS, OS, and predictive factors

**Fig 2:**
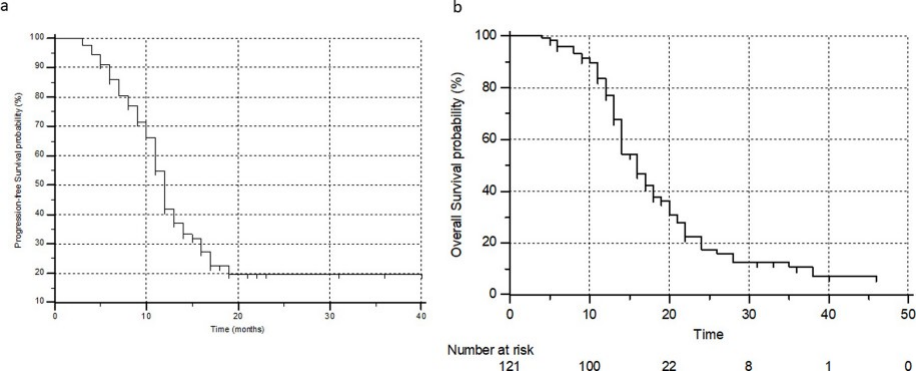
**a.** Progression-free survival according to PCWG3 criteria, **b.** overall survival.

**Fig 3:**
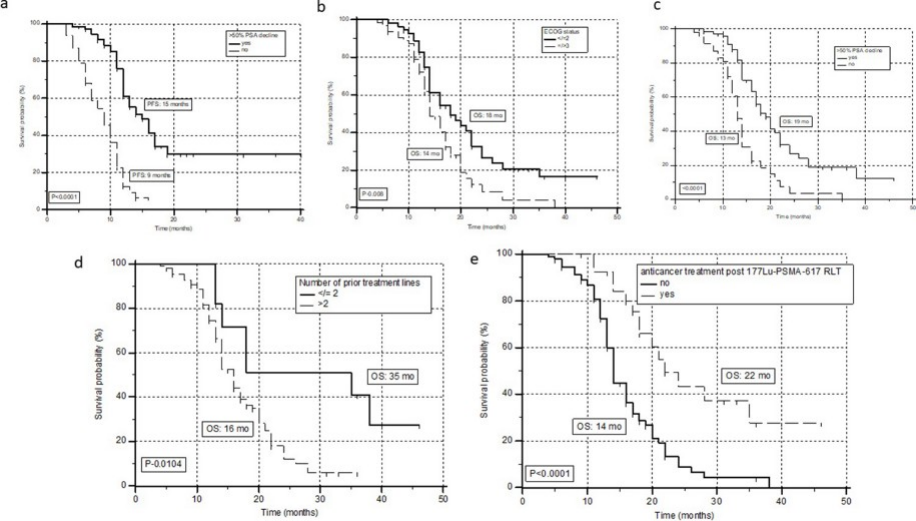
Kaplan-Meier plots of prognostic factors associated with PFS and OS. **a.** Kaplan-Meier plots of progression-free survival comparing the patient cohort wit >50% PSA decline vs. no decline. **b-e.** Kaplan-Meier curves of factors such as ECOG status (cut-off: 2), >50% PSA decline, number of prior systemic treatment lines of treatment (cut-off: 2), and further anti-cancer treatment post ^177^Lu-PSMA-617 RLT associated with the overall survival.

During the follow-up, 79 (65%) patients experienced disease progression with a median PFS of 12 mo (95% CI: 10.3–13), and a 24-months progression-free survival probability of 19.7% (Fig 2A). Thirty-eight patients in this series, who experienced >50% PSA decline during the treatment, eventually demonstrated disease progression during the follow-up period. The univariate analysis revealed no prior history of androgen-inhibitor treatment chemotherapy naïve, patients experiencing any PSA decline and/or >50% PSA decline as significant predictors of prolonged PFS (Table 2). However, multivariate, stepwise, Cox proportional hazard regression analysis revealed only failure to demonstrate more than 50% PSA decline (hazard ratio [HR], 4.1; P<0.0001; 95% CI: 2.584–6.650) as significant factors associated with an inferior PFS. (Table 2) (Fig 3A).

**Table 2:** Univariate and multivariate Cox proportional hazard regression of factors associated with progression-free survival.

	Univariate Analysis			Multivariate Analysis	
Parameters	Median PFS (mo)	P (Log-rank)	HR; 95% CI	P	HR; 95% CI
**Age (years)**					
<70	12	0.092	0.7 (0.444–1.078)		
≥ 70	12				
**Gleasons score**					
<7	12	0.805	1.0 (0.629–1.805)		
≥7	12				
**Primary/residual disease**					
No	12	0.804	1. (0.648–1.703)		
Yes	12				
**Lymph node metastases**					
No	12	0.842	0.9 (0.593–1.541)		
yes	12				
**Lung metastases**					
No	12	0.642	0.8 (0.384–1.793)		
Yes	12				
**Liver metastases**					
No	12	0.814	1.9 (0.379–2.144)		
Yes	11.5				
**Number of bone metastases**					
≤ 20	12	0.602	0.9 (0.507–1.512)		
>20	12				
**ECOG performance status**					
0–2	12	0.672	1.09 (0.704–1.701)		
≥ 3	12				
**ALP values (U/L)**					
≤ 333	12	0.990	1 (0.637–1.594)		
>333	12				
**Chemotherapy**					
No	12	0.148	1.6 (0.901–2.900)		
Yes	12				
**Number of lines of chemotherapy**					
No	12	0.014	1.7 (1.123–2.871)		
One line of Cx	12				
Two or more lines of Cx	10				
**Androgen inhibitor therapy**					
No	14	0.016	1.3 (1.063–1.824)		
History of treatment	11				
**Second-generation anti androgen**					
No	12	0.219	1.3 (0.766–2.431)		
Yes	11				
**Any PSA Decline**					
No	7	<0.0001	3.4 (1.827–6.207)		
Yes	13				
**PSA decline >50%**					
No	9	<0.0001	3.6 (2.136–6.091)	<0.0001	4.1 (2.584–6.650)
Yes	15				
**Number of previous standard lines of treatment**					
≤ 2	17	0.084	1.9 (1.036–3.7108)		
>2	12				
**Concomitant therapies**					
No	10	0.150	1.4 (0.817–2.441)		
Yes	12				

PFS: Progression-free survival; HR: Hazards ratio; 95% CI: 95% confidence interval; ECOG: Eastern cooperative oncology group performance status; ALP: alkaline phosphatase values; PSA: Prostate-specific antigen.

At the time of analysis, 83 (68.5%) patients were deceased. The median OS was 16 mo (95% CI: 13–17 mo) irrespective of response to treatment with a 12 and 24-months survival probability of 77% and 18%, respectively (Fig 2B).

On univariate analysis, ECOG performance status ≤2, chemotherapy naïve, any PSA decline, >50% PSA decline, patients who were pre-treated with ≤2 lines of standard anti-cancer systemic treatment, and patients who were further subjected to anti-cancer treatment post-^177^Lu-PSMA-617 therapy were associated with significantly prolonged overall survival (Table 3). While, multivariate analysis revealed only failure to experience >50% PSA decline [HR: 2.5; 95% CI: 1.697–3.939; P<0.0001] (Fig 3C), heavily pre-treated patients with >2 lines of prior standard treatment options [HR: 2.9; 95% CI: 1.207–7.012; P-0.01] and patients who received more than one-line of chemotherapy [HR: 1.5; 95% CI: 1.018–2.321; P-0.041] were remarkably associated with poor OS (Fig 3D). Interestingly, the multivariate analysis also revealed that patients who were further treated after ^177^Lu-PSMA-617 therapy with second-line and salvage treatment options showed a significantly prolonged survival with the least hazards ratio of 0.3; 95% CI: 0.164 – 0.550; P-0.0001 (Fig 3E) (Table 3).

**Table 3:** Univariate and multivariate Cox-proportional hazard regression of factors associated with overall survival.

	Univariate Analysis			Multivariate Analysis	
Parameters	Median OS (mo)	P (Log-rank)	HR; 95% CI	P	HR; 95% CI
**Age (years)**					
<70	16	0.431	0.8 (0.547–1.306)		
≥ 70	18				
**Gleasons score**					
<7	16	0.891	1.03 (0.617–1.735)		
≥7	16				
**Primary/residual disease**					
No	18	0.884	1.03 (0.648–1.646)		
Yes	16				
**Lymph node metastases**					
No	17	0.797	1.05 (0.673–1.660)		
yes	16				
**Lung metastases**					
No	16	0.550	0.7 (0.343–1.730)		
Yes	14				
**Liver metastases**					
No	16	0.917	1.04 (0.447–2.427)		
Yes	14				
**Number of bone metastases**					
≤ 20	19	0.694	0.6 (0.198–1.881)		
>20	18				
**ECOG performance status**					
0–2	18	0.008	1.7 (1.094–2.637)		
≥ 3	14				
**ALP values (U/L)**					
≤ 333	16	0.621	1.1 (0.717–1.712)		
>333	14				
**Chemotherapy**					
No	22	0.032	2 (1.198–3.651)		
Yes	16				
**Number of lines of chemotherapy**					
No		0.033	1.6 (1.041–2.482)	0.041	1.5 (1.018–2.321)
One line of Cx					
Two or more lines of Cx					
**Androgen inhibitor therapy**					
No	16	0.585	0.9 (0.700–1.221)		
History of treatment	18				
Ongoing treatment	17				
**Second-generation anti androgen**					
No	16	0.335	0.7 (0.415–1.298)		
Yes	18				
**Any PSA Decline**					
No	13	0.001	2 (1.164–3.458)		
Yes	17				
**PSA decline >50%**					
No	13	<0.0001	2.5 (1.525–4.053)	0.0001	2.5 (1.697–3.939)
Yes	19				
**PSA progression >25%**					
Yes	14	0.250	1.3 (0.826–2.097)		
No	17				
**Number of previous standard lines of treatment**					
≤ 2	35	0.010	2.4 (1.352–4.154)	0.017	2.9 (1.207–7.012)
>2	16				
**Concomitant therapies**					
No	16	0.880	1 (0.618–1.741)		
Yes	16				
**Anti-cancer treatment after** ^**177**^**Lu-PSMA-617 therapy**					
No	14	<0.0001	0.4 (0.224–0.549)	0.0001	0.3 (0.164–0.550)
Yes	22				

OS: Overall survival; HR: Hazards ratio; 95% CI: 95% confidence interval; ECOG: Eastern cooperative oncology group performance status; ALP: Alkaline Phosphatase; PSA: Prostate-specific antigen.

On a detailed analysis of the various regimes of treatment that were opted in patients after either on progression during ^177^Lu-PSMA-617 therapy or during the follow-up period after completion of ^177^Lu-PSMA-617 therapy cycles, 27 patients underwent further salvage treatment options that are detailed in Table 4. Interestingly, 13 of these 27 patients are still alive and are responding to their current treatments (Table 4). A sub-categorical analysis revealed a remarkably improved OS in these patients treated with alternative treatment options even after progression on ^177^Lu-PSMA-617 therapy compared to those who did not receive further treatment. [14 vs. 22 mo, HR: 0.4, 95% CI: 0.224–0.549; P<0.0001] with consistent significance in the multivariate Cox-proportional hazard model (Fig 3E).

**Table 4:** Details of patients treated with alternate anti-cancer treatment options after ^177^Lu-PSMA-617 RLT.

S. No	Age	Number of cycles	Cumulative activity (mCi)	Survival status	OS duration	Time to progression on ^177^LU-PSMA-617 therapy	Treatment after ^177^Lu-PSMA-617
1	79	4	600	Alive	-	12	^225^Ac-PSMA-617 4 cycles (complete response)
2	62	3	360	Death	24	8	^225^Ac-PSMA-617 4 cycles (Progression after 12 mo followed by death)
3	49	3	450	Death	17	10	^225^Ac-PSMA-617 4 cycles (Progression after 12 mo followed by death
4	70	2	300	Alive	-	11	^225^Ac-PSMA-617 (partial response)
5	65	4	700	death	12	12	Re-challenged Docetaxel
6	38	6	700	Death	28	17	Enzalutamide 4 mo (Progressive disease followed by death)
7	57	3	600	Death	35	12	Docetaxel 3 cycles (progression), Cabazitaxel 2 cycles (Progression)
8	72	5	270	Death	18	14	Abiraterone acetate (Progression after 2 mo followed by death)
9	69	7	940	Alive	-	17	Docetaxel 4 cycles (progression), Enzalutamide 4 mo (progression), ^225^Ac- PSMA-617 (ongoing, complete response)
10	59	4	600	Alive	-	13	Docetaxel 3 cycles (progression) Cabazitaxel 3 cycles (progression) ^225^Ac-PSMA-617 (ongoing, partial response)
11	75	7	1010	Alive	-	30	^225^Ac-PSMA-617 (Complete response)
12	62	6	1000	Alive	-	19	Abiraterone acetate (ongoing, partial response)
13	64	6	1000	Alive	-	14	Enzalutamide 2 mo (progression) ^225^Ac-PSMA-617 (near-complete response)
14	62	4	600	Death	24	13	^225^Ac-PSMA-617 (4 cycles followed by disease progression after 8 mo followed by death)
15	55	3	280	Death	14	11	Enzalutamide 2 mo (progression and death)
16	78	6	980	Death	27	15	Enzalutamide 2 mo (progression) Cabazitaxel 1 cycle (stopped due to AE) ^225^Ac-PSMA-617 2 cycles (death)
17	81	4	486	Death	21	13	^225^Ac-PSMA-617 (progression after 4 cycles and death)
18	78	6	800	Death	20	12	Enzalutamide, Cabazitaxel, ^225^Ac-PSMA-617
19	72	4	600	Death	22	12	Enzalutamide ^225^Ac-PSMA-617
20	65	5	750	Alive	-	12	Abiraterone (partial response)
21	71	2	240	Death	14	5	^225^Ac-PSMA-617 (2 cycles, progression, death)
22	62	3	450	Death	16	12	^225^Ac-PSMA-617 (2 cycles, partial response, death)
23	72	4	600	Alive	-	7	^225^Ac-PSMA-617 (2 cycles, stable disease)
24	69	2	300	Alive	-	9	^225^Ac-PSMA-617 (4 cycles, partial response)
25	78	3	300	Alive	-	10	Enzalutamide
26	70	2	300	Alive	-	7	^225^Ac-PSMA-617 (2 cycles partial response)
27	51	2	250	Alive	-	4	Cabazitaxel 2 cycles (progression) ^225^Ac-PSMA-617 (ongoing, stable disease)

### Biochemical PSA response assessment

During the treatment and follow-up, 88 (73%) patients experienced any PSA decline, and the best PSA response of >50% PSA decline was seen in 74 (61%) patients.

During the treatment or follow-up, >25% PSA progression was observed in, 79 (65%) patients. Among them, 38 patients had shown >50% PSA response during some point of the treatment and follow-up.

### Molecular response

Interim and the follow-up molecular response assessment could be assessed in 105 patients. Three patients achieved complete response during the treatment and remained disease-free till their end-follow-up. Out of 27 patients who demonstrate initial PR, two achieved complete remission, eight patients further responded to treatment (NCR/PR), and 17 patients progressed. Thirty-three patients demonstrated molecular disease progression at their first interim ^68^Ga-PSMA PET/CT scan and did not respond to further treatment (Table 5).

**Table 5:** Molecular response findings at during the ^177^Lu-PSMA RLT and at last follow-up.

Interim findings on ^68^Ga-PSMA PET/CT scan (N)	^68^Ga-PSMA PET/CT findings at the last follow-up (N)
CR- 3	CR- 3
SD - 42	PD - 28
	PR - 1
	SD - 13
PR - 27	CR - 2
	NCR - 5
	PR - 3
	PD - 17
PD- 33	PD- 33
NOT ASSESSED - 16	

N: Number of patients; CR: complete response; PR: Partial response; NCR: Near complete response; PD: Progressive disease

### Clinical response

There was a significant reduction in the VASmax score post-treatment (8 vs. 5; P < 0.0001). Similarly, a significant decrease in mean AS from the baseline was observed (3 vs. 2 ± 1 [P< 0.000]). The median KPS and ECOG status significantly improved from 60 to 70 (P < 0.0001) and 3 to 2 (P = 0.0005), respectively.

### Toxicity and adverse events

The most common side effect from ^177^Lu-PSMA-617 treatment was grade I/II fatigue (42/121 [34.7%]) that lasted up to 2 wks post-RLT and nausea (40/121 [33%]) persisted for 3–4 days post-RLT. Dry mouth was experienced in 31/121 (24.7%) of patients; however, it was limited to grade I/II and was transient, but recurred after the subsequent cycle of ^177^Lu-PSMA-617 RLT. they were transient and limited to grade I/II only. Other toxicities such as diarrhea were reported in 14% 18/121) of patients. Grade III anemia was noted in three patients. Interestingly, there was no grade III thrombocytopenia or leukopenia in any of the patients. Regarding nephrotoxicity, two patients with grade I kidney toxicity at the baseline marginally increased to grade II toxicity, and no higher-grade toxicity was observed (Table 6).

**Table 6:** Adverse events, according to CTCAE v5.0.

Baseline						After radioligand therapy				
Event	Grade 0	Grade 1	Grade 2	Grade 3	Grade 4	Grade 0	Grade 1	Grade 2	Grade 3	Grade 4
Anemia	6	104	11	0	0	0	80	38	3	0
Thrombocytopenia	106	14	1	0	0	91	28	2	0	0
Leukopenia	119	0	2	0	0	99	20	2	0	0
Creatinine level increased	96	25	0	0	0	111	8	2	0	0
Xerostomia	0	0	0	0	0	96	23	2	0	0

WBC: White blood cell counts; CTCAE: Common toxicity criteria for adverse events

## Discussion

This study aimed to give an overview on the long-term outcome of ^177^Lu-PSMA-617 therapy in a heavily pre-treated mCRPC patient cohort, who had received at least two lines of prior treatment; particularly, focused on the survival outcomes and the prognostic factors that influence the survival. In this study, we present a single institutional 6-year experience by highlighting the long-term outcome of ^177^Lu-PSMA-617 therapy.

To the best of our knowledge, there is only one study by Violet et al. [4] and ours would be the second to elaborate the long-term outcome of patients treated with ^177^Lu-PSMA-617 RLT with a median follow-up duration of 36 months. Similarly, both the studies narrate the response and pattern of disease progression in patients during treatment and post-treatment follow-up. However, while we studied the outcome of ^177^Lu-PSMA-617 therapy where patients were further treated with various systemic salvage therapeutic options, Violet et al. [4] re-challenged the second set of ^177^Lu-PSMA-617 therapy regimen in their patient cohort. In this current study, we report the pattern of progression, the time to disease progression after the initial response, and the further course of treatment with additional systemic anti-cancer/salvage treatment options.

Violet et al. [4] reported a >50% PSA decline rate of 64% when re-treated with ^177^Lu-PSMA-617 RLT, which is well in line with the PSA response rate observed in the current study in 61% of patients. The long-term PSA response rates concur well with both previously published retrospective and prospective short-term results [1]. However, when comparing our results to those of older studies, it must be pointed out that the PSA response rates were in the lower range. A recently published meta-analysis in 744 mCRPC patients on ^177^Lu-PSMA-617 reported a pooled >50% PSA decline rate of 46% (95% CI, 40–53%) [1].

The primary outcome measure in this study was the overall survival. Violet et al. [4] observed a median OS of 13.3 mo (95% CI: 10.5–8.5) compared to 16 mo in our study. In agreement, meta-analysis data pooled from the previously published works addressed a median overall survival of 13.7 (IQR: 8–14) mo [1]. Moreover, the long-term OS data were similar to and consistent with the short-term survival data.

The secondary objective of our study was to identify the prognostic factors influencing overall survival. The association of PSA response with the overall survival has been previous studied with variable results. We observed that a PSA response of >50% is a significant predictor of prolonged OS. A similar result was reported by Violet et al. [4]. However, in short-term studies, the impact of >50% PSA decline on the OS remains inconsistent with few studies favoring it as a critical prognostic indicator of OS, whereas some other studies contradicting the fact [12, 13, 15–17]. This inconsistency in data leads to an interesting finding that a PSA response at the initial phase does not always translate to a longer OS in mCRPC patients with aggressive metastases. In contrast, both long-term studies concurred that PSA response of >50% decline predicts prolonged OS. Our findings on OS contradict the results of Brauer et al. [13] and Ahmadzadehfar et al. [12]. However, a later study by Ahmadzadehfar et al. [14] reported a decline in PSA levels of >50% significantly attributed to the longer median OS (70 wks; 95% CI: 39.5–100.5) compared those patients with PSA decline <50% (49 wks; 95% CI: 30.2–67.8). The discrepancy in the two results may be due to the selection bias, small sample size, heterogeneity in the disease burden, and probably shorter duration of follow-up (Table 7).

**Table 7:** Studies reporting the factor predicting the OS and PFS.

Study	N	Median follow-up duration	Median no of cycles	Median OS	Median PFS	Factors predicting OS univariate	Factors predicting OS multivariate	Factors predicting PFS univariate	Factors predicting PFS multivariate
Ahmadzadefar et al. (2017) [12]	100	At least 2 mo	3	60 wks (95% CI: 47.3–72.7)	-	Number of bone metastases, existence of liver metastases, ECOG status, blood transfusion before the first cycle, GGT (cut-off: 31 U/l), ALP (cut-off: 140 U/l), PSA any decline, PSA decline ≥ 50%, a regular need for analgesics regular need for opioids, PSA percent change (cut-off: - 14%), Albumin (cut-off: 38.6 g/l), AST (cut-off: 24 U/l)), Hb (cut-off: 10.4 g/dl), PSA percent change (cut-off: - 14%),	Existence of liver metastases, Albumin (cut-off: 38.6 g/l) 0.01 AST (cut-off: 24 U/l) 0.04 Hb (cut-off: 10.4 g/dl), PSA percent change (cut-off: –14%)	-	-
Bräuer et al. (2017) [13]	59	24 wks (IQR 15–36).	3 (1–7)	32.0 wks (95% CI 21.1–42.9)	18.0 wks (95% CI 13.6–22.4)	any initial PSA decline, ALP value <220 U/L	No factors associated	ALP levels <220 U/L	ALP levels <220 U/L
Ahmadzadefar et al. (2017} [14]	52	The median follow-up time after the last cycle was 4.5 mo (2–12)	(3–6)	60 wks in all patients irrespective of response (95%CI: 44.2–75.8	-	Any PSA decline PSA, decline of ≥50%	-	-	-
Rahbar et al. (2018) [15]	104	-	3 (1–8)	56.0 wks (95%CI: 50.5–61.5		Any initial PSA decline, initial ALP <220 U/L, injected activity ≥18.8 GBq	PSA decline ≥20.87%	-	-
Heck et al. (2019) [16]	100	Only alive patients 9.5 mo (IQR: 7.0–16.3)	2 (1–6)	12.9 mo	4.1	Primary mCRPC, visceral metastasis, age, PSA, increasedLDH	LDH, Visceral metastasis	Clinical PFS: visceral metastasis age, LDH	Visceral metastasis, age, LDH
Kessel et al. (2017) [17]	54	-	3	9.9 mo (95% CI: 7.2–12.5)	-	Any initial decline of PSA, second line cabazitaxel, visceral metastases	Only visceral metastases	-	-
Yadav et al. (2019) [5]	90	28 mo (6–35)	4 (1–7)	14 mo (95% CI: 13–16 mo)	11 mo (95% CI, 9–13 mo)	ECOG performance status, ALP (cut-off: 240 U/L), any PSA progression, PSA decline (>50%), PSA decline (on first Follow-up)	ECOG performance status, PSA decline (>50%)	-	-
Violet et al. (2020) [4]	50	31.4 mo	Initial (2–4) Re-treatment (1–5)	13.3 mo (95% CI 10.5–18.7)	6.9 mo (95% CI 6.0–8.7)	PSA decline ≥ 50%	-	(psaPFS) PSA decline ≥ 50%	-
Ahmadzadehfar et al. (2020) [18]	416 (multi- centre)	Minimum 6 mo follow-up	3 (1–12)	11.1	-	Prior chemotherapy, existence of bone and liver metastases, ECOG status	Prior chemotherapy, existence of bone and liver metastases, ECOG status	-	-

OS: overall survival; PFS: progression-free survival; ECOG: Eastern cooperative oncology group performance status; CI: confidence interval; ALP: alkaline phosphatase; PSA: Prostate-specific antigen; wks: weeks

One of the key findings from this study indicates that the overall survival remarkably improved in the patient cohort who were eventually treated with other anti-cancer treatment options after the discontinuation of ^177^Lu-PSMA-617 RLT. These findings correlate well with Violet et al. [4] and further favours the concept of re-challenging patients with other advanced salvage treatment options after acquiring radio-resistance on ^177^Lu-PSMA-617 RLT.

### Influence of prior lines of treatment on OS

An important observation noted in the current study is that patients who were subjected to more than two prior systemic lines of treatment options showed poor overall survival. In this context, Ahmadzadehfar et al. [18] retrospectively evaluated the impact of prior therapies on OS in 416 patients treated with ^177^Lu-PSMA-617 from 11 different clinics. The multicentre analysis revealed prior chemotherapy as a significant independent adverse prognostic factor for poor overall survival. Similar to the findings of Ahmadzadehfar et al. [18], both univariate and multivariate analyses demonstrated prior treatment with >2 lines of therapies, and a prior history of 2 lines of chemotherapy significantly reduced OS. Similarly, Kessel et al. [17] observed second-line cabazitaxel chemotherapy (6.7 vs. 15.7 mo, P = 0.002) significantly associated with a shorter OS compared to patients who had not received second-line chemotherapy (7.9 vs.14.6 mo, Log-rank P = 0.002; HR 2.1, P = 0.009). From the above data, it is clear that heavily pre-treated patients with multiple lines of treatment negatively impact the overall survival (Table 7).

### Influence of concomitant treatment on OS

Several clinical trials on concomitant treatment with ^177^Lu-PMSA-617 are in progress. Although a prior anti-hormonal therapy with either abiraterone or enzalutamide or both was not a significant predictive factor, there was a significant difference between the OS of patients with a history of enzalutamide and patients who were under concurrent usage of enzalutamide during ^177^Lu-PSMA-617 treatment (12.3 vs. 10.8 mo, respectively; P = 0.045)

Combination therapy, especially regarding second-line androgen deprivation therapy or chemotherapy, was assessed in our patient population, and its use did not affect the OS in the long-run. The short-term results of our previous study also demonstrated concomitant medication prolonged the survival but could not remain a predictive factor on multivariate analysis. However, these findings are preliminary, and the type of concomitant medications varied across the patients. But definitive clinical trials are in progress to evaluate the impact of concomitant treatment on ^177^Lu-PSMA-617 therapy.

The most awaited among the trials include the VISION phase III trial comparing ^177^Lu-PSMA-617 vs. best supportive or best standard of care in mCRPC patients (NCT03511664). The ANZUP TheraP clinical trial is active and underway, which compares ^177^Lu-PSMA-617 vs. Cabazitaxel in progressive mCRPC (NCT03392428). Prospective RCTs comparing ^177^Lu-PSMA-617 with Olaparib (PARP inhibitor, recently FDA approved) and immunotherapy (PD-1 and PD-L1 inhibitors), respectively, are in the pipeline (NCT03874884) and (NCT03658447).

### Re-treatment cohort

Twenty-seven patients with disease progression further underwent other lines of treatment, among which 16 patients primarily received ^225^Ac-PSMA-617 therapy and have shown remarkable response rate. Fifty-six percent of the patients are alive and have shown improved overall survival. Recently, a study on ^225^Ac-PSMA-617 in mCRPC patients from our group has shown a promising disease control-rate (DCR), and minimal treatment-related toxicities in patients refractory to ^177^Lu-PSMA-617 therapy [19]. Eight patients also received enzalutamide among whom 3 are alive (Table 4). The impact of further treatment post after ^177^Lu-PSMA-617 may be the underlying fact for improved survival of 22 mo compared to 14 mo in the re-treatment naïve cohort.

### Treatment-related toxicities

The long-term follow-up of these patients after the completion of ^177^Lu-PSMA-617 RLT will best answer the delayed complications. The results of our study reassure the earlier findings of short-term observations regarding the safety of RLT; the toxicities were minimal and rare chances of delayed toxicity to the critical organs such as the kidneys and salivary glands. There is no adverse risk noted over several years of meticulous follow-up of these patients to the dose-limiting organs. Similarly, a study on the toxicity of the re-challenged patient cohort with ^177^Lu-PMSA-617 reported no unexpected adverse events with ^177^Lu-PSMA-617 retreatment [4]. Keeping this in mind, higher fixed doses of administered activity per cycle and/or re-challenge of ^177^Lu-PSMA-617 therapy regimen may be seriously considered. In the future, even administering concurrent therapies in these patients is feasible. ^177^Lu-PSMA-617 therapy is safe with limited toxicities, even in heavily pre-treated mCRPC patients with extensive bone-marrow involvement. The pancytopenia was limited to grade 2, transient with a nadir around four weeks of therapy. Less serious, but more common, side effects included xerostomia, fatigue, and nausea.

### Limitations

The study suffers from certain limitations. Firstly, it’s a retrospective and single-center study. The study population was heterogeneous with a wide range of ECOG performance status, variation in the pattern of metastases and, heterogeneities in the prior therapies due to the broad inclusion criteria. Hence the treatment protocol was individualized and may suffer from heterogeneity in the treatment and outcomes. Genomic data was not assessed and hence, a clinico-genetic correlation was out of the scope of this paper.

## Conclusion

From this long-term follow-up study, we conclude that ^177^Lu-PSMA-617 RLT is an effective form of therapy in mCRPC patients and confirms the findings of the short-term studies. ^177^Lu-PSMA-617 prolongs the overall survival in mCRPC patients heavily pre-treated with chemotherapy, first and second-line anti-androgens, and androgen inhibitor therapies. The delayed toxicity is low and acceptable by most of these patients. Patients who were treated with various other mCRPC-approved compounds before ^177^Lu-PSMA-RLT were associated with a significantly shorter OS than that of the patients who received ^177^Lu-PSMA RLT at the earlier stages of mCRPC. An aggressive treatment approach, either sequential or in combination with second-generation anti-androgens, chemotherapies, or with investigational ^225^Ac-PSMA-617 alpha therapies, might prolong the survival of mCRPC patients in the future.

## Supporting information

S1 File (XLSX)

## References

[1] Yadav MP, Ballal S, Sahoo RK, Dwivedi SN, Bal C. Radioligand Therapy With 177Lu-PSMA for Metastatic Castration-Resistant Prostate Cancer: A Systematic Review and Meta-Analysis. AJR Am J Roentgenol. 2019; 21: 275–285. 10.2214/ajr.18.20845 30995089

[2] Hofman MS, Violet J, Hicks RJ, Ferdinandus J, Thang SP, Akhurst T, et al. [177Lu]-PSMA-617 radionuclide treatment in patients with metastatic castration-resistant prostate cancer (LuPSMA trial): a single-centre, single-arm, phase 2 study. Lancet Oncol. 2018; 19: 825–833. 10.1016/s1470-2045(18)30198-0 29752180

[3] Calais Jeremie, Gartmann Jeannine, Armstrong Wesley R, Thin Pan, Nguyen Kathleen, Lok Vincent, et al. 10.1200/JCO.2020.38.15_suppl.5549 Journal of Clinical Oncology 38, 2020, (Suppl 15): 5549–5549. Published online May 25, 2020.) ClinicalTrials.gov Identifier: NCT03042312.

[4] Violet J, Sandhu S, Iravani A, Ferdinandus J, Thang SP, Kong G, et al. Long-Term Follow-up and Outcomes of Retreatment in an Expanded 50-Patient Single-Center Phase II Prospective Trial of 177Lu-PSMA-617 Theranostics in Metastatic Castration-Resistant Prostate Cancer. J Nucl Med. 2020; 61: 857–865. 10.2967/jnumed.119.236414 31732676 PMC7262220

[5] Yadav MP, Ballal S, Bal C, Sahoo RK, Damle NA, Tripathi M, et al. Efficacy and Safety of 177Lu-PSMA-617 Radioligand Therapy in Metastatic Castration-Resistant Prostate Cancer Patients. Clinical Nuclear Medicine. 2020; 45: 19–31. 10.1097/rlu.0000000000002833 31789908

[6] Yadav MP, Ballal S, Tripathi M, Damle NA, Sahoo RK, Seth A, et al. Post-therapeutic dosimetry of 177LuDKFZ-PSMA-617 in the treatment of patients with metastatic castration-resistant prostate cancer. Nucl Med Commun. 2017; 38: 91–98. 10.1097/mnm.0000000000000606 27782913

[7] Scher HI, Morris MJ, Stadler WM, Higano C, Basch E, Fizazi K, et al. Trial Design and Objectives for Castration-Resistant Prostate Cancer: Updated Recommendations From the Prostate Cancer Clinical Trials Working Group 3. J Clin Oncol. 2016; 34: 1402–1418. 10.1200/jco.2015.64.2702 26903579 PMC4872347

[8] Wahl RL, Jacene H, Kasamon Y, Lodge MA. From RECIST to PERCIST: evolving considerations for PET response criteria in solid tumors. J Nucl Med. 2009; 50: S122–150. 10.2967/jnumed.108.057307 19403881 PMC2755245

[9] McCaffery M & Pasero C. Pain: Clinical Manual. 2^nd^ ed. St Louis, MO: Mosby; 1999. 10.1046/j.1365-2702.2000.0374c.x.

[10] Crooks V, Waller S, Smith T, Hahn TJ, The use of the Karnofsky Performance Scale in determining outcomes and risk in geriatric outpatients, J Gerontol. 46 (1991) 139–144. 10.1093/geronj/46.4.m139 2071835

[11] Common Terminology Criteria for Adverse Events (CTCAE) v5.0 Publish Date: November 27, 2017.

[12] Ahmadzadehfar H, Schlolaut S, Fimmers R, Yordanova A, Hirzebruch S, Schlenkhoff C et al. Predictors of overall survival in metastatic castration-resistant prostate cancer patients receiving 177Lu-PSMA-617 radioligand therapy. Oncotarget. 2017; 8: 103108–103116. 10.18632/oncotarget.21600 29262549 PMC5732715

[13] Bräuer A, Grubert LS, Roll W, Schrader AJ, Schäfers M, Bögemann M et al. (177) Lu-PSMA-617 radioligand therapy and outcome in patients with metastasized castration-resistant prostate cancer. Eur J Nucl Med Mol Imaging. 2017; 44: 1663–1670. 10.1007/s00259-017-3751-z 28624848

[14] Ahmadzadehfar H, Wegen S, Yordanova A, Fimmers R, Kürpig S, Eppard E et al. Overall survival and response pattern of castration-resistant metastatic prostate cancer to multiple cycles of radioligand therapy using [(177)Lu]Lu-PSMA-617. Eur J Nucl Med Mol Imaging. 2017; 44: 1448–1454. 10.1007/s00259-017-3716-2 28488028

[15] Rahbar K, Boegemann M, Yordanova A, Eveslage M, Schafers M, Essler M, et al. PSMA targeted radioligand therapy in metastatic castration resistant prostate cancer after chemotherapy, abiraterone and/or enzalutamide. A retrospective analysis of overall survival. Eur J Nucl Med Mol Imaging. 2018; 45: 12–19. 10.1007/s00259-017-3848-4 29026946

[16] Heck MM, Tauber R, Schwaiger S, Retz M, D’Alessandria C, Maurer T, et al. Treatment Outcome, Toxicity, and Predictive Factors for Radioligand Therapy with (177)Lu-PSMA-I&T in Metastatic Castration-resistant Prostate Cancer. Eur Urol. 2019; 75: 920–926. 10.1016/j.eururo.2018.11.016 30473431

[17] Kessel K, Seifert R, Schäfers M, Weckesser M, Schlack K, Boegemann M, et al. Second line chemotherapy and visceral metastases are associated with poor survival in patients with mCRPC receiving 177Lu-PSMA-617. Theranostics. 2019; 9: 4841–4848. 10.7150/thno.35759 31410185 PMC6691377

[18] Ahmadzadehfar H, Rahbar K, Baum RP, Seifert R, Kessel K, Bögemann M, et al. Prior therapies as prognostic factors of overall survival in metastatic castration-resistant prostate cancer patients treated with [177Lu]Lu-PSMA-617. A WARMTH multicenter study (the 617 trial). Eur J Nucl Med Mol Imagin. 2021; 48: 113–122. 10.1007/s00259-020-04797-9. PMC7835179 32383093

[19] Yadav MP, Ballal S, Sahoo RK, Tripathi M, Seth A, Bal C. Efficacy and safety of 225Ac-PSMA-617 targeted alpha therapy in metastatic castration-resistant Prostate Cancer patients. Theranostics. 2020; 10: 9364–9377. 10.7150/thno.48107 32802197 PMC7415797

